# High-performance liquid chromatography–tandem mass spectrometry for simultaneous determination of 23 antidepressants and active metabolites in human serum and its application in therapeutic drug monitoring

**DOI:** 10.3389/fphar.2025.1531496

**Published:** 2025-03-27

**Authors:** Jinglong Wang, Junjie Wang, Chenxiao Zhang, Guofei Li

**Affiliations:** Department of Pharmacy, Shengjing Hospital of China Medical University, Shenyang, China

**Keywords:** HPLC, tandem mass spectrometry, validation, antidepressants, TDM

## Abstract

**Introduction:**

The incidence and mortality rate from depression are increasing year by year, and depression has become the main cause of global health loss and disability. Currently, the treatment of depression mainly relies on drug intervention. However, the vast majority of antidepressants exhibit significant pharmacological variability, resulting in individual differences in steady-state blood drug concentrations even with the same dosing regimen among patients. Therefore, using therapeutic drug monitoring (TDM) to guide the precise use of antidepressants has important clinical significance.

**Methods:**

In this paper, we developed a high-performance liquid chromatography–tandem mass spectrometry (HPLC–MS/MS) method to study simultaneously TDM and clinical pharmacokinetics of 23 antidepressants and active metabolites: sertraline, escitalopram, fluvoxamine, paroxetine, duloxetine, milnacipran, fluoxetine, venlafaxine, O-desmethylvenlafaxine, mirtazapine, trazodone, bupropion, hydroxybupropione, norfluoxetine, vortioxetine, agomelatine, mianserin, doxepine, desmethyldoxepin, clomipramine, desmethylclomipramine, amitriptyline and nortriptyline hydrochloride. After protein precipitation of serum samples with acetonitrile, the isotope internal standards (ISs), antidepressants and active metabolites were separated using a ZORBAX Eclipse Plus C18 column (50.0 mm × 2.1 mm, 1.7 µm) with water containing 0.1% formic acid and 10 mmol/L ammonium acetate and methanol containing 0.1% formic acid. Validation of the developed method was carried out based on the Chinese Pharmacopoeia guidelines for bioanalytical method validation, including assessment of specificity, calibration curves, carryover, accuracy, crosstalk, precision, stability, recovery, dilution integrity and matrix effect.

**Results:**

The results showed that a simple, fast, reliable and specific HPLC'MS/MS method was developed and validated, and all the performance characteristics of the method met the requirements, which could be used to study TDM and pharmacokinetics of the above 23 antidepressants and active metabolites.

## 1 Introduction

Major depressive disorder (MDD) refers to a type of disease characterized by significant and persistent low mood caused by various reasons (Schramm et al., 2020; Kang and Cho, 2020). Depression is the most common type of MDD, characterized by low mood, loss of interest and lack of energy (Shorey et al., 2021; Rotenstein et al., 2016). Depression has the characteristics of high incidence, high recurrence and high disability. If it cannot be treated in a timely and effective manner, it will lead to a huge social and economic burden. Depressive symptoms often do not receive sufficient attention from patients, family members and doctors, and depression associated with most physical illnesses is easily overlooked (Monroe and Harkness, 2022). The treatment and intervention rates for self-injury, suicide and drug and alcohol dependence caused by depression are even lower (Shorey et al., 2021). According to the World Health Organization’s projections, by 2030, the disease burden of depression will surpass ischemic cardiomyopathy and become the world’s leading disease burden (Vollset et al., 2020). Therefore, how to treat depression efficiently and accurately has become an urgent problem to be solved (Monroe and Harkness, 2022; Herrman et al., 2019).

The treatment methods for depression mainly include medication and psychological therapy, with medication being the main treatment (Kverno and Mangano, 2021; Cipriani et al., 2018). There are various types of antidepressants, including new and traditional antidepressants. Traditional antidepressants include: (1) tricyclic antidepressants (Undurraga and Baldessarini, 2017): mainly exert antidepressant effects by inhibiting the reuptake of 5-HT and NE by the presynaptic membrane and increasing the concentration of 5-HT and NE in the synaptic cleft. Representative drugs include amitriptyline and doxepin. (2) Monoamine oxidase inhibitors (Corbineau et al., 2017): inhibit the metabolic enzymes of monoamine neurotransmitters, causing an increase in the concentration of monoamine neurotransmitters in the synaptic cleft, such as metoclopramide. The new antidepressants include mainly: (1) selective serotonin reuptake inhibitors, which can selectively inhibit the uptake of 5-HT by the presynaptic membrane and increase the concentration of 5-HT in the synaptic cleft (Latendresse et al., 2017). They are first-line antidepressants, including fluoxetine, paroxetine, fluoxamine, sertraline, citalopram and escitalopram, (2) serotonin and norepinephrine reuptake inhibitors: can simultaneously inhibit the reuptake of 5-HT and NE by the presynaptic membrane (Strawn et al., 2023). Representative drugs include venlafaxine, duloxetine and milnacipran. (3) Noradrenergic and specific serotonergic antidepressants: can antagonize central presynaptic α2 self-receptors and alloreceptors, increase NE and 5-HT release and their neural conduction, such as mirtazapine (Kessing et al., 2024). (4) Norepinephrine and dopamine reuptake inhibitors: the representative drug is bupropion, which has a weak inhibitory effect on NE and DA reuptake (Wang et al., 2023). However, its active metabolite is a strong reuptake inhibitor and has a high concentration in the brain. (5) Multimodal antidepressants: mainly related to increased serotonin activity caused by inhibition of 5-HT reuptake, represented by the drug vortioxetine (Ceskova, 2016). (6) Serotonin antagonists and reuptake inhibitors: can antagonize 5-HT2A receptors and inhibit the reuptake of 5-HT by presynaptic membranes (Arias et al., 2021). The representative drug is trazodone. (7) NMDA receptor antagonist: ketamine, a representative drug, has a rapid antidepressant effect (Rajkumar et al., 2015). Overall, these drugs are effective and essential for the treatment of depression, but the efficacy for many patients does not satisfy doctors and patients. According to statistics, 38% of patients with depression do not respond to antidepressants (Wyska, 2019), resulting in a low overall effectiveness rate of depression treatment. Therefore, while focusing on new drug development, it may be more meaningful to explore the factors that affect the efficacy of antidepressant drugs.

The clinical efficacy and adverse reactions of antidepressants are closely related to the patient’s blood drug concentration level. However, the vast majority of antidepressants exhibit significant pharmacological variability, especially pharmacokinetic variability, resulting in significant individual differences in steady-state blood drug concentration and efficacy even with the same dosing regimen among patients (Maslej et al., 2021; Radosavljevic et al., 2023; Wyska, 2019). The reasons may include: 1) patient factors: patients with depression are more varied, and the vast majority of patients require long-term or even lifelong medication. Poor medication adherence has become the primary factor affecting drug concentration. In addition, special populations with comorbid depression, such as the elderly, children and pregnant women, are prone to drastic fluctuations in blood drug concentrations due to changes in renal blood flow, clearance rate and hormone levels. 2) Disease factors: depression often presents multiple types, with complex subtypes that are easy to transform into each other. Therefore, accurate diagnosis of depression has always been a difficult problem for clinical doctors. 3) Drug interactions: According to statistics, approximately half of patients with depression require combination therapy. Research has shown that the interaction between antidepressants induced by combination therapy has become another key factor affecting blood drug concentration. 4) Changes in laboratory indicators, such as protein levels and liver and kidney function, can cause significant changes in drug concentrations that are metabolized by the liver, excreted by the kidneys and have high protein binding rates, ultimately affecting therapeutic efficacy. 5) Genetic polymorphism: the vast majority of antidepressants are metabolized and transported by hepatic enzymes and transporters, and the genetic polymorphism of metabolic enzymes and transporters is closely related to the concentration of their substrates. 6) Selection of TDM indicators: most antidepressants use steady-state trough concentration as the TDM indicator, but some drugs have metabolites with significant pharmacological activity. So, it may be more meaningful to evaluate the relationship between drugs and efficacy using the total concentration of the parent drug and active metabolites, such as fluoxetine, bupropion and venlafaxine. Therefore, we should fully recognize the gap between existing pharmacological knowledge and its clinical application, and “precision therapy” may be the key to bridging this gap.

TDM, the main technical means to guide precise clinical drug use, is the use of modern analytical methods to determine the concentration of drugs or their metabolites in blood or other body fluids (Fiaturi and Greenblatt, 2019). Sample preparation methods play a crucial role in accurate TDM results. Commonly used sample preparation methods for drug analysis in TDM include liquid-liquid extraction (LLE), solid-phase extraction (SPE), and protein precipitation. LLE is a classic method that uses the difference in solubility of substances in two immiscible solvents to separate the target analyte (Patteet et al., 2015). SPE, on the other hand, is more selective and efficient, using solid sorbents to retain the analyte from the sample matrix. Protein precipitation is a relatively simple method, which is often used when rapid sample processing is required. Each method has its own advantages and limitations, and the choice of method depends on various factors such as the nature of the drug, the matrix of the sample, and the detection method.

Antidepressants can be determined not only in blood but also in other biological materials such as urine, saliva, and cerebrospinal fluid. Analyzing antidepressants in urine can provide information about recent drug intake (Taghizadeh et al., 2022), and saliva sampling is non-invasive, which is more convenient for patients, especially in pediatric or geriatric populations (Dziurkowska and Wesolowski, 2023). Cerebrospinal fluid is rather special. Its acquisition is an invasive procedure, and it is not a conventional matrix or body fluid like blood, urine, and saliva. Thus, it is generally not used as a routine test sample. Nevertheless, cerebrospinal fluid analysis can provide insights into the drug concentration in the central nervous system, which is directly related to the therapeutic effect of antidepressants (Moaddel et al., 2022).

By applying the principles of pharmacokinetics and pharmacodynamics, TDM guides clinical drug treatment, individualizes patient dosing regimens, improves efficacy and reduces adverse reactions. As for antidepressants, there are large individual differences and narrow safety ranges, requiring individualized administration. Therefore, guiding the precise use of such drugs through TDM has important clinical significance (Piacentino et al., 2022; Hiemke et al., 2017). Although China began to develop TDM for antidepressant drugs in the 1990s, the development was slow. Therefore, there is still a significant degree of arbitrariness in the medication used by patients with depression in China, resulting in poor clinical efficacy. There are many reasons for this situation, among which the most important one is the lack of TDM methods with high accuracy and wide applicability. Therefore, with the continuous application of new antidepressant drugs in clinical practice, the development of new TDM methods is an urgent problem to be solved.

Currently, in general clinical practice, especially in primary medical institutions and large-scale screening scenarios, the *in-vivo* concentration detection of antidepressants is often carried out using immunological methods. These methods, such as chemiluminescent immunoassays, are favored for their simplicity, rapid operation, and relatively low cost. They can quickly provide results, which is beneficial for basic clinical diagnosis and initial treatment decision-making (National Center for Clinical Laboratories of the National Health Commission in China, 2024).

However, for antidepressants with extensive pharmacokinetic characteristics in *in-vivo* metabolism, the anti-interference ability of immunological methods is relatively limited. This leads to a slightly lower accuracy compared to more advanced chromatographic methods like liquid chromatography - tandem mass spectrometry (LC - MS/MS), which has become the “gold standard” for *in vivo* drug analysis (Tuzimski and Petruczynik, 2020; Li et al., 2022). Considering the demand for highly accurate results in in-depth research and some complex clinical cases, there is an urgent need to develop methods with strong anti-interference ability to better meet the requirements of precise therapeutic drug monitoring.

While the HPLC-MS/MS method we developed, which uses MS/MS detection, is not a cost-effective approach, it has distinct advantages. It can simultaneously detect the serum drug concentrations of 23 commonly used antidepressants and their active metabolites with strong specificity, good stability, high sensitivity, and an appropriate retention time. This high-precision detection ability enables more accurate TDM, which is crucial for optimizing the treatment of depression. In the future, research could focus on exploring ways to optimize the cost-effectiveness of this method without sacrificing its high-performance capabilities, such as streamlining the sample preparation process or finding more cost-effective reagents. This would not only expand the application scope of this method but also promote the development of more precise antidepressant treatment strategies.

To sum up, we developed an HPLC–MS/MS method to study simultaneously TDM and clinical pharmacokinetics of antidepressants and active metabolites: sertraline, escitalopram, fluvoxamine, paroxetine, duloxetine, milnacipran, fluoxetine, venlafaxine, O-desmethylvenlafaxine, mirtazapine, trazodone, bupropion, hydroxybupropione, norfluoxetine hydrochloride, vortioxetine, agomelatine, mianserin, doxepine, desmethyldoxepin, clomipramine, desmethylclomipramine, amitriptyline and nortriptyline hydrochloride. Validation of the developed HPLC–MS/MS method was carried out based on the Chinese Pharmacopoeia guidelines for bioanalytical method validation. The results showed that a simple, fast, reliable and specific HPLC–MS/MS method was developed and validated, and all the performance characteristics of the method met the requirements, which could be used to study TDM and pharmacokinetics of the above 23 antidepressants and active metabolites, which will provide a theoretical basis for the standardization and widespread development of antidepressant drug TDM.

## 2 Methods

### 2.1 Experimental reagents

Sertraline, escitalopram, fluvoxamine, paroxetine, duloxetine, milnacipran, fluoxetine, venlafaxine, O-desmethylvenlafaxine, mirtazapine, trazodone, bupropion, hydroxybupropione, norfluoxetine hydrochloride, vortioxetine, agomelatine, mianserin, doxepine, desmethyldoxepin, clomipramine, desmethylclomipramine, amitriptyline, nortriptyline hydrochloride and ISs (sertraline-d3, citalopram-d6, fluvoxamine-d3, paroxetine-d4, duloxetine-d7, milnacipran-d5, fluoxetine-d5, venlafaxine-d6, O-desmethylvenlafaxine-d3, hydroxybupropione-d6, mirtazapine-d3, trazodone-d4, bupropion-d9, norfluoxetine-d5, vortioxetine-d8, agomelatine-d6, mianserin-d3, desmethylclomipramine-d3, desmethyldoxepin-d3, doxepine-d3, clomipramine-d3, amitriptyline-d6 and nortriptyline-d4) were purchased from Tianjin Alta Technology Co., Ltd. HPLC-grade methanol was purchased from Fisher Scientific (Fair Lawn, NJ, United States). HPLC-grade formic acid was obtained from Sigma-Aldrich. The pure water for HPLC analysis was obtained using a Milli-Q water purification system (Millipore Corp., United States).

### 2.2 Equipment and conditions

The Jasper™ HPLC system (Shimadzu, Japan), equipped with a SCIEX Dx Controller, SCIEX Dx Sampler, SCIEX Dx Pump (×2), SCIEX Dx Degasser, SCIEX Dx Oven and a dual 108-well plate autosampler, was used for the chromatography analysis, facilitating efficient and reproducible injection of samples into the chromatographic system and ensuring high precision and accuracy throughout the process. The ISs and 23 antidepressants and active metabolites were separated using a ZORBAX Eclipse Plus C18 column (50.0 mm × 2.1 mm, 1.7 μm) with water containing 0.1% formic acid and 10 mmoL ammonium acetate and methanol containing 0.1% formic acid. The column temperature was set to 40°C, as this temperature was found to provide optimal separation of the compounds under investigation, based on preliminary method development experiments. The sample injection volume was 2.0 μL.

The MS spectrometric detection of the ISs, 23 antidepressants and active metabolites was carried out on a SCIEX Triple Quad 4500MD System with an electrospray ionization detector. A SCIWAY BIO-ABN nitrogen generator was used to prepare high-purity nitrogen gas for MS. The main parameters such as ionization mode, transition, collision energy, declustering potential, dwell time, collision cell exit potential and spray voltage are shown in Table 2. The remaining parameters were as follows: ion source temperature 450°C, CAD medium, entrance potential 15 V, curtain gas 40 psi, GS1 50 psi and GS2 40 psi. Analyst software v1.6.2, which comes with the MS system, was used to process the data.

### 2.3 Stock solutions, quality control samples and calibration standards

Standard solutions (stock solution, work solution and calibration solution) of all the analytes were dissolved in methanol at 500.0 μg/mL and stored at −70°C. A series of concentration standard solutions was prepared by diluting the above stock solution in methanol. Working mixture solutions of the 23 antidepressants and active metabolites were obtained and mixed by dilution in methanol based on the concentrations of the drugs in serum at their recommended therapeutic concentration range. For the calibration curve, working solutions of the mixed standards were obtained by mixed and continuously diluting the stock solution at six concentration levels: 4, 16, 40, 160, 400 and 1,000 ng/mL for sertraline, mirtazapine, vortioxetine, agomelatine, bupropion, mianserin, escitalopram, paroxetine and duloxetine; 10, 40, 100, 400, 1,000 and 2,500 ng/mL for fluoxetine, norfluoxetine hydrochloride, fluvoxamine, clomipramine, desmethylclomipramine, desmethyldoxepin, venlafaxine, O-desmethylvenlafaxine, doxepine, milnacipran, amitriptyline and nortriptyline hydrochloride; 20, 80, 200, 800, 2,000 and 5,000 ng/mL for hydroxybupropione and trazodone.

The final working solutions of quality controls (QCs) were prepared at four concentrations: lower limit of quantitation (LLOQ), QC low (LQC), QC medium (MQC) and QC high (HQC) at concentrations of 4, 8, 200 and 800 ng/mL for sertraline, mirtazapine, vortioxetine, agomelatine, bupropion, mianserin, escitalopram, paroxetine and duloxetine; 10, 20, 500 and 2,000 ng/mL for fluoxetine, norfluoxetine, desmethyldoxepin, fluvoxamine, O-desmethylvenlafaxine, desmethylclomipramine, venlafaxine, clomipramine, doxepine, milnacipran, amitriptyline and nortriptyline hydrochloride; 20, 40, 1,000 and 4,000 ng/mL for hydroxybupropione and trazodone. All QC samples were stored at −70°C until further use.

IS working solutions of 100 ng/mL for sertraline-d3, mirtazapine-d3, vortioxetine-d8, agomelatine-d6, bupropion-d9, mianserin-d3, citalopram-d6, paroxetine-d4 and duloxetine-d7; 200 ng/mL for fluoxetine-d5, norfluoxetine-d5, fluvoxamine-d3, clomipramine-d3, desmethylclomipramine-d3, desmethyldoxepin-d3, venlafaxine-d6, nortriptyline-d4, amitriptyline-d6, doxepine-d3, milnacipran-d5 and O-desmethylvenlafaxine-d3; 400 ng/mL for hydroxybupropione-d6 and trazodone-d4 were obtained by diluting stock solution with methanol. Except for escitalopram which used citalopram-d6 as the internal standard, all other analytes used their respective isotopes as the internal standard.

### 2.4 Plasma samples

All serum samples were stored at −70°C and thawed at room temperature before being processed for HPLC–MS/MS analysis. The protein precipitation (PPT) method was used to extract the 23 antidepressants and active metabolites from human serum as follows: 250 µL of acetonitrile (containing all the ISs) was added to 50 µL of patient serum, spiked serum (40 µL of analyte-free human plasma and 10 µL of standard solutions) or blank (40 µL of analyte-free human serum and 10 µL of MeOH) samples and vortex-mixed for 1 min. After vortex mixing, the samples were then centrifuged at 15,000 g for 5.0 min at 4°C and 200 μL of 8% methanol (initial proportion of mobile phase) was added to 30 μL supernatant. After thorough vortexing for 1.0 min, the solutions were injected into the HPLC–MS/MS system for analysis.

### 2.5 Method validation

The developed analytical method was validated according to the Chinese Pharmacopoeia guidelines for bioanalytical method validation (Pharmacopoeia Commission of the People’s Republic of China, 2020; United States Food and Drug Administration, 2018).

#### 2.5.1 Sensitivity and specificity

The sensitivity of the HPLC–MS/MS method was assessed by preparing the LLOQ from six different human serums and determining the signal-to-noise ratio, which was set at an eligible limit of higher than 10. By contrast, the specificity of the method was evaluated by extracting blank plasma from six different sources to check for coeluting peaks at the retention times of the analytes.

#### 2.5.2 Linearity, LLOQ and carryover

Standard curves were obtained by plotting the peak area ratio of analyte and IS against the corresponding analyte concentration, and the linearity of the plot was assessed by evaluating three standard curves on three consecutive days. The concentration range was 4–1,000 ng/mL for sertraline, mirtazapine, vortioxetine, agomelatine, bupropion, mianserin, escitalopram, paroxetine and duloxetine; 10–2,500 ng/mL for fluoxetine, norfluoxetine hydrochloride, fluvoxamine, clomipramine, desmethylclomipramine, desmethyldoxepin, venlafaxine, O-desmethylvenlafaxine, doxepine, milnacipran, amitriptyline and nortriptyline hydrochloride; 20–5,000 ng/mL for hydroxybupropione and trazodone. The influence of carryover on the measurement results was evaluated by determining a blank sample after the upper limit of quantitation (ULOQ), and the carryover should be <±20% of LLOQ.

#### 2.5.3 Accuracy and precision

Precision and accuracy were expressed as relative standard deviation (RSD%) and relative error (RE%, the difference between the average value and the true value of the QC sample), respectively. In this paper, precision and accuracy were evaluated by assessing six repeated measurements of the blood samples at LLOQ, LQC, MQC and HQC on three consecutive days. The RSD and RE should be less than ±15%, whereas the acceptance criterion was no more than ±20% for LLOQ.

#### 2.5.4 Extraction recovery and matrix effect

Extraction recovery and matrix effect of analytes were assessed in four different samples at the concentrations of LLOQ, LQC, MQC and HQC. Extraction recovery was calculated by comparing the peak area of the extracted sample to that of blanks spiked with analytes postextraction. The matrix effect of endogenous substances was investigated by comparing the peak areas of each antidepressant or active metabolite that existed in extracted blank plasma with the peak areas of each antidepressant or active metabolite diluted in pure water. The precision of the QCs at each concentration was set to be within 15%, and no more than 20% for LLOQ.

#### 2.5.5 Stability

The stability of all the analytes in human serum was determined by analyzing the human QC samples under different conditions, including autosampler stability (24 h, 4°C), short-term stability (12 h, room temperature), freeze–thaw stability (three freeze–thaw cycles, from −20.0°C to room temperature) and long-term storage stability (30 days, −70°C).

#### 2.5.6 Dilution integrity

We assessed dilution integrity by diluting plasma samples higher than ULOQ with blank plasma to the HQC levels. The dilution factor was set to 10 and 50 times, which covers more than 99% of clinical samples (except for a few peak concentration points). The criteria were deemed satisfied when the precision and the accuracy were less than ±15%.

### 2.6 Application

In this study, we used the established HPLC–MS/MS method to monitor the steady-state trough concentration of 23 antidepressants and active metabolites, to guide individualized clinical medication. We determined 40 sertraline, 25 escitalopram, 30 fluvoxamine, 19 paroxetine, 28 milnacipran, 30 fluoxetine, 59 venlafaxine, 34 mirtazapine, 65 trazodone, 31 bupropion, 51 vortioxetine, 44 agomelatine, 35 doxepine, 68 clomipramine, 53 amitriptyline and 26 nortriptyline hydrochloride using the proposed HPLC–MS/MS method.

Briefly, clinical blood samples were collected from those patients who were taking the above antidepressant drugs in the period between April 2023 and April 2024. The blood sample was first centrifuged at 4,000 g for 5 min to obtain a serum sample, and then processed according to the serum sample processing method. We used the clinical data of patients with depression with the permission of the patients. The required healthy blank human plasma was provided by the Hematology Department of Shengjing Hospital of China Medical University.

## 3 Results and discussion

### 3.1 Method optimization

**FIGURE 1:**
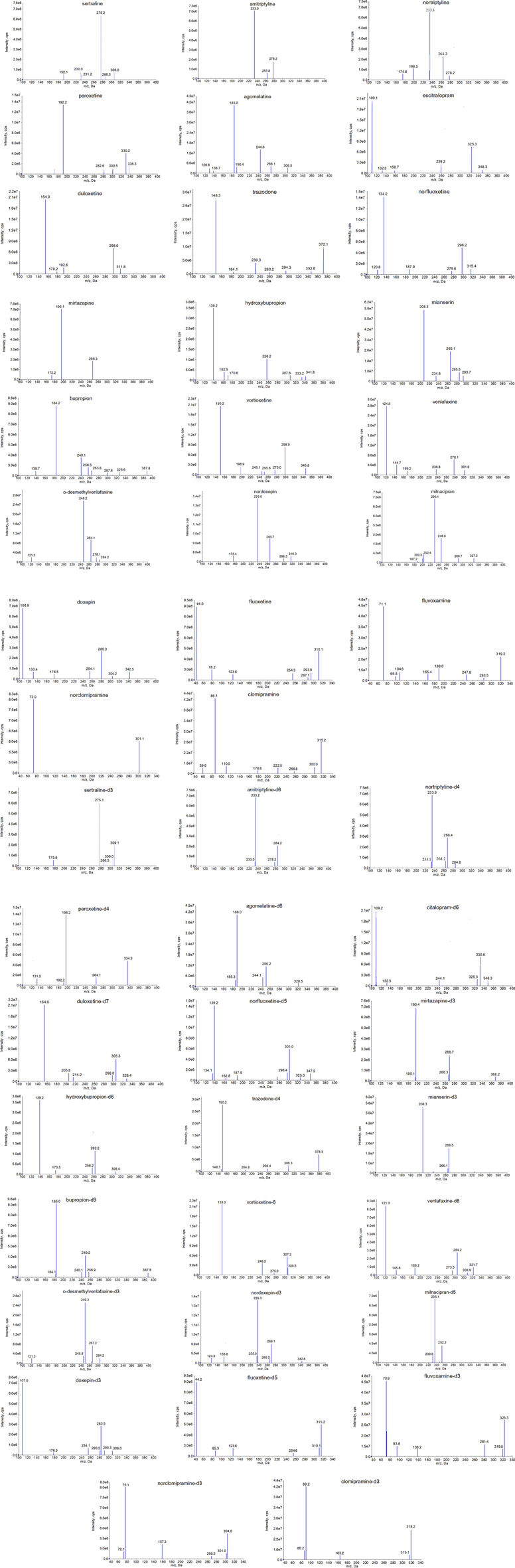
The mass spectra of daughter scan of 23 antidepressants and active metabolites.

A qualified HPLC–MS/MS method capable of simultaneously determining multiple drugs should have the following characteristics: 1) appropriate retention time, sharp and symmetrical chromatographic peaks and low residual effects; 2) strong specificity, high sensitivity, minimal matrix effects and no interference between the analytes. To achieve this goal, we systematically optimized the mobile phase composition (water, methanol, acetonitrile), the types of mobile phase additives (different concentrations of formic acid, acetic acid, ammonium formate and ammonium acetate), chromatographic column (ZORBAX Eclipse Plus C18 column (50.0 mm × 2.1 mm, 1.7 μm), Agilent Eclipse XDB-C18 column (50.0 mm × 2.1 mm, 1.7 μm), Poroshell 120 EC-C18 (4.6 mm × 100 mm, 2.7 µm) and Venusil XBP C18 (4.6 mm × 150 mm, 5 µm)) and elution mode (isocratic elution and gradient elution). After optimizing the chromatographic conditions, we found that a ZORBAX Eclipse Plus C18 column (50.0 mm × 2.1 mm, 1.7 μm) column with a mobile phase composition of water containing 0.1% formic acid and 10 mmoL ammonium acetate and methanol containing 0.1% formic acid can yield sharp, symmetrical and well separated chromatographic peaks for each analyte and IS. The final HPLC–MS/MS conditions are shown in Tables 1, 2. As for MS conditions, all analytes and ISs can achieve stronger and more stable signals in positive ion ionization mode than in negative mode, the MS parameters and mass spectra of final ion pairs are shown in Table 2; Figure 1. The present research also assessed liquid–liquid extraction (ether, dichloromethane, ethyl acetate, etc.) and protein precipitation (methanol, acetonitrile, 15% perchloric acid) for the extraction efficiency of analytes from serum. Finally, simple protein precipitation with acetonitrile can obtain good recovery and low matrix effect for all the analytes and ISs.

**TABLE 1:** Gradient condition of HPLC.

Time (min)	A (%)^a^	B (%)^b^	Flow rate (mL/min)
Initial	92	8	0.6
0.60	92	8	0.6
0.61	50	50	0.6
1.50	30	70	0.6
1.60	2	98	0.6
2.50	2	98	0.6
2.51	92	8	0.6
3.00	92	8	0.6

^a^ Water containing 0.1% formic acid and 10 mmoL ammonium acetate.

^b^ Methanol containing 0.1% formic acid.

**TABLE 2:** MS parameters of analytes and ISs.

	Transition (m/z)	Collision cell exit potential(V)	Collision energy (V)	Declustering potential (s)	Spray voltage (V)	Dwell time (msec)
Sertraline	306.0→275.2	10	18	30	4,500	15
Escitralopram	325.3→109.1	22	35	100	4,500	15
Fluvoxamine	319.2→71.1	20	32	60	4,500	35
paroxetine	330.2→192.2	14	30	100	4,500	15
duloxetine	298.0→154.0	11	7	50	4,500	20
milnacipran	246.8→230.1	7	16	95	4,500	35
fluoxetine	310.1→44.0	8	40	75	4,500	30
Venlafaxine	278.1→121.0	8	38	90	4,500	25
o-desmethylvenlafaxine	264.1→246.2	10	17	60	4,500	30
Mirtazapine	266.3→195.1	20	32	80	4,500	20
trazodone	372.1→148.3	20	45	110	4,500	15
bupropion	240.1→184.2	12	17	60	4,500	20
Hydroxybupropion	256.2→139.2	8	33	50	4,500	10
Norfluoxetine	296.2→134.2	10	11	50	4,500	10
mianserin	265.1→208.3	10	27	90	4,500	10
Nordoxepin	265.7→235.0	10	21	70	4,500	10
Doxepin	280.3→106.9	7	28	90	4,500	10
agomelatine	244.0→185.0	11	21	96	4,500	10
Vortioxetine	298.9→150.2	11	31	110	4,500	10
Clomipramine	315.2→86.1	40	22	130	4,500	10
Amitriptyline	278.2→233.0	10	24	80	4,500	10
Nortriptyline	264.2→233.3	13	22	85	4,500	10
Norclomipramine	301.1→72.0	40	46	100	4,500	10
sertraline-d3	309.1→275.1	10	18	30	4,500	15
citalopram-d6	330.6→109.2	22	35	100	4,500	15
fluvoxamine-d3	323.3→70.9	20	32	60	4,500	35
paroxetine-d4	334.3→196.2	14	30	100	4,500	15
duloxetine-d7	305.3→154.0	11	7	50	4,500	20
milnacipran-d5	252.2→235.1	7	16	95	4,500	35
fluoxetine-d5	315.2→44.2	8	40	75	4,500	30
venlafaxine-d6	284.2→121.3	8	38	90	4,500	25
o-desmethylvenlafaxine-d3	267.2→249.3	10	17	60	4,500	30
mirtazapine-d3	268.7→195.4	20	32	80	4,500	20
trazodone-d4	378.3→150.2	20	45	110	4,500	15
bupropion-d9	249.2→185.0	12	17	60	4,500	20
hydroxybupropion-d6	262.2→139.2	8	33	50	4,500	10
norfluoxetine-d5	301.0→139.2	10	11	50	4,500	10
mianserin-d3	268.5→208.3	10	27	90	4,500	10
nordoxepin-d3	269.1→235.3	10	21	70	4,500	10
doxepin-d3	283.5→107.0	7	28	90	4,500	10
agomelatine-d6	250.2→188.0	11	21	96	4,500	10
vortioxetine-d8	307.2→153.0	11	31	110	4,500	10
clomipramine-d3	318.2→89.2	40	22	130	4,500	10
amitriptyline-d6	284.2→233.2	10	24	80	4,500	10
nortriptyline-d4	268.4→233.9	13	22	85	4,500	10
norclomipramine-d3	304.0→75.1	40	46	100	4,500	10

### 3.2 Method validation

#### 3.2.1 Sensitivity and specificity

**FIGURE 2:**
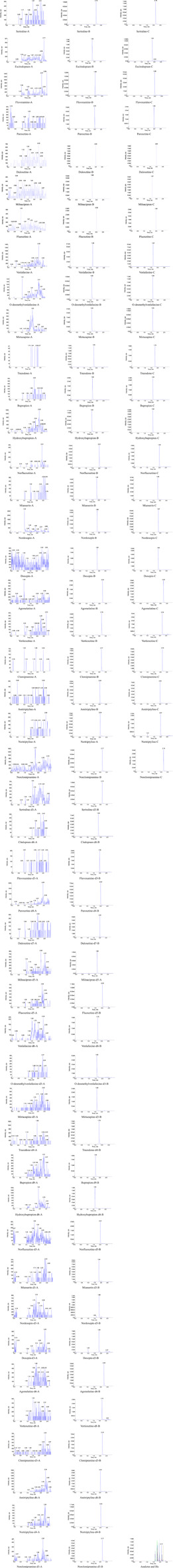
Representative HPLC-MS/MS chromatograms for antidepressants and active metabolites in human serum samples: **(A)** a blank plasma sample **(B)** a blank plasma sample spiked with analytes and IS, and **(C)** plasma sample of patients (Analytes and ISs) chromatograms of all analytes and ISs.

Considering that the established method is mainly used for clinical TDM, the LLOQ for all analytes in this study is not the lowest value. Therefore, the signal-to-noise ratio is much greater than 10, and the sensitivity can fully meet the requirements. In terms of specificity, as shown in the chromatogram of blank human serum and the chromatogram of the analyte in human serum (Figure 2), the developed HPLC–MS/MS method has good specificity for all analytes, and no coeluting peak of endogenous substances was observed at the retention time of the analyte. Sertraline, escitalopram, fluvoxamine, paroxetine, duloxetine, milnacipran, fluoxetine, venlafaxine, O-desmethylvenlafaxine, mirtazapine, trazodone, bupropion, hydroxybupropione, norfluoxetine hydrochloride, vortioxetine, agomelatine, mianserin, doxepine, desmethyldoxepin, clomipramine, desmethylclomipramine, amitriptyline and nortriptyline hydrochloride had a retention time of 2.15, 1.82, 1.98, 2.00, 2.00, 1.67, 2.02, 1.70, 1.60, 1.63, 1.80, 1.66, 1.63, 2.01, 2.19, 2.24, 1.85, 1.85, 1.86, 2.17, 2.17, 2.03 and 2.04 min, respectively.

#### 3.2.2 Linearity, LLOQ and carryover

The linearity of the calibration standards was assessed on three separate occasions. All the analytes in human serum provided good linearity over the concentration range of 4–1,000 ng/mL for sertraline, mirtazapine, vortioxetine, agomelatine, bupropion, mianserin, escitalopram, paroxetine and duloxetine; 10–2,500 ng/mL for fluoxetine, norfluoxetine hydrochloride, fluvoxamine, clomipramine, desmethylclomipramine, desmethyldoxepin, venlafaxine, O-desmethylvenlafaxine, doxepine, milnacipran, amitriptyline and nortriptyline hydrochloride; 20–5,000 ng/mL for hydroxybupropione and trazodone, with correlation coefficients (*r*) not less than 0.9979. The accuracy of the calibration standards for all the analytes in human serum was within 100% ± 7.8%, with a CV (%) of ≤8.2%. The LLOQ of sertraline, mirtazapine, vortioxetine, agomelatine, bupropion, mianserin, escitalopram, paroxetine and duloxetine was 4 ng/mL, fluoxetine, norfluoxetine hydrochloride, fluvoxamine, clomipramine, desmethylclomipramine, desmethyldoxepin, O-desmethylvenlafaxine, venlafaxine, doxepine, milnacipran, amitriptyline and nortriptyline hydrochloride was 10 ng/mL and hydroxybupropione and trazodone was 20 ng/mL, the corresponding chromatograms are provided in the Supplementary Material. In addition, the crosstalk between the analytes and ISs was completely negligible.

#### 3.2.3 Accuracy and precision

As shown in Table 3, the intraday precision for all the analytes was found in the range 1.6%–9.1%, whereas the interday precision ranges were from 0.8% to 10.9%. Meanwhile, the accuracy was obtained in the range of −7.2%–11.6%. Therefore, the proposed method had good precision and accuracy.

**TABLE 3:** Methodology verification results of precision, extraction recovery and matrix effect.

Drug	QC concentration (μg/mL)	Intra-day		Inter-day		Recovery (Mean ± SD)	Matrix effect (Mean ± SD)
		RSD %	RE %	RSD %	RE %		
sertraline	4	5.2	2.9	4.9	−3.8	94.7 ± 4.6	98.2 ± 4.5
	8	4.6	4.2	3.4	4.9	96.3 ± 5.3	97.1 ± 3.3
	200	3.3	3.5	2.0	3.4	93.6 ± 4.2	99.7 ± 2.8
	800	5.1	−2.5	2.4	−2.7	98.3 ± 5.1	91.2 ± 0.6
mirtazapine	4	7.4	−2.9	3.7	−2.6	102.4 ± 3.4	97.8 ± 4.1
	8	6.2	4.5	4.6	4.9	99.0 ± 4.2	94.9 ± 1.4
	200	1.9	−3.8	3.3	3.9	95.7 ± 1.6	93.6 ± 2.5
	800	3.8	−4.9	2.9	−4.7	98.2 ± 2.9	95.0 ± 4.7
vortioxetine	4	4.0	3.47	3.9	−3.3	96.5 ± 4.6	104.2 ± 3.6
	8	4.9	3.5	2.3	3.6	91.7 ± 2.6	97.4 ± 2.0
	200	6.4	4.0	4.9	4.2	93.4 ± 3.0	102.8 ± 5.7
	800	2.6	−1.7	5.8	−5.3	96.6 ± 4.2	107.4 ± 3.3
agomelatine	4	9.1	2.3	10.9	4.9	97.2 ± 3.9	99.4 ± 4.5
	8	5.5	4.9	8.3	−3.6	101.8 ± 4.7	96.3 ± 2.1
	200	3.6	5.4	6.1	4.4	100.3 ± 5.3	102.4 ± 3.6
	800	2.5	3.2	2.0	3.8	95.5 ± 2.6	100.7 ± 2.6
bupropion	4	4.2	−1.6	2.6	5.2	93.6 ± 1.5	97.2 ± 5.2
	8	4.5	−5.5	4.5	−5.8	97.0 ± 3.8	99.9 ± 4.5
	200	3.6	3.1	4.8	2.7	92.1 ± 3	97.7 ± 1.7
	800	5.0	−2.6	5.7	−3.9	97.2 ± 7	92.9 ± 4.6
mianserin	4	2.7	4.6	3.4	4.2	99.4 ± 4.9	94.1 ± 3.0
	8	3.2	2.4	5.9	4.4	93.7 ± 2.4	96.5 ± 3.7
	200	3.6	−2.3	6.4	−2.5	97.6 ± 1.0	97.4 ± 3.1
	800	2.9	−4.1	4.7	−3.5	98.5 ± 5.3	98.9 ± 4.7
escitalopram	4	7.3	3.7	3.2	2.2	92.1 ± 3.6	94.8 ± 4.2
	8	4.5	3.6	4.6	3.9	97.5 ± 4.1	91.7 ± 2.8
	200	3.0	1.6	3.2	−2.8	96.5 ± 3.8	97.0 ± 2.5
	800	2.2	3.3	4.7	1.4	98.4 ± 2.7	93.1 ± 3.1
paroxetine	4	5.1	−3.8	4.9	−3.6	104.6 ± 5.3	98.0 ± 4.4
	8	3.6	−2.5	5.5	−2.3	93.8 ± 4.9	96.3 ± 3.9
	200	4.8	3.9	1.7	−3.8	103.8 ± 3.4	97.5 ± 1.7
	800	2.6	2.7	2.6	4.0	95.5 ± 4.0	98.2 ± 2.9
duloxetine	4	7.7	−4.2	3.6	2.9	101.3 ± 3.1	95.6 ± 4.6
	8	5.3	3.6	2.8	−3.2	98.3 ± 2.6	96.8 ± 3.8
	200	1.6	3.5	2.9	1.6	94.2 ± 4.8	96.6 ± 3.4
	800	3.9	2.3	3.1	−5.5	95.1 ± 5.2	99.4 ± 3.6
fluoxetine	10	2.2	−2.4	3.0	−2.6	96.0 ± 3.7	103.7 ± 1.0
	20	3.6	0.9	3.7	3.6	92.9 ± 2.0	91.0 ± 1.7
	500	2.7	2.9	4.5	3.5	96.4 ± 5.5	95.6 ± 2.2
	2000	5.3	−3.2	2.2	5.2	106.5 ± 4.7	98.3 ± 4.0
norfluoxetine	10	3.7	−2.7	6.6	−1.7	94.6 ± 4.9	92.5 ± 3.8
	20	4.7	4.3	7.7	3.3	96.2 ± 5.1	98.2 ± 2.7
	500	8.0	2.5	5.6	−2.6	96.8 ± 3.7	95.8 ± 3.5
	2000	3.8	4.1	3.7	4.8	98.9 ± 5.2	100.4 ± 1.8
	10	5.8	−1.9	2.3	9.4	94.2 ± 3.3	102.0 ± 2.6
desmethyldoxepin	20	2.5	2.6	3.8	−3.2	97.4 ± 3.6	97.3 ± 3.9
	500	3.1	−3.7	3.9	−4.1	99.6 ± 2.7	97.6 ± 4.1
	2000	4.7	−2.6	4.2	5.0	99.1 ± 2.9	104.6 ± 3.6
	10	5.8	7.3	5.6	4.5	91.6 ± 1.6	99.5 ± 4.4
hydrochloride	20	3.2	−6.3	4.3	−2.7	96.5 ± 3.8	96.8 ± 3.2
	500	4.1	2.8	5.7	5.3	98.2 ± 4.2	97.4 ± 1.9
	2000	2.7	4.0	2.7	−2.8	95.9 ± 4.4	99.6 ± 2.8
	10	2.9	5.8	1.9	3.5	102.3 ± 4.9	94.6 ± 3.6
nortriptyline	20	1.4	−6.3	4.5	−1.8	103.6 ± 3.5	96.5 ± 5.0
	500	1.8	2.5	4.2	−3.0	99.0 ± 5.2	102.6 ± 4.6
	2000	2.2	3.8	5.8	4.8	100.4 ± 4.6	103.7 ± 5.5
	10	4.1	4.1	0.8	5.7	97.1 ± 3.5	95.8 ± 2.6
hydrochloride	20	3.9	−1.5	3.5	7.8	98.4 ± 2.7	97.4 ± 3.7
	500	2.7	3.3	4.1	4.3	96.6 ± 4.3	99.3 ± 4.2
	2000	2.0	4.5	2.8	−3.4	92.6 ± 1.5	96.9 ± 2.6
	10	6.2	−3.9	4.7	2.6	98.5 ± 1.9	108.2 ± 3.1
doxepine	20	5.8	−7.2	5.4	−5.5	96.4 ± 4.3	100.5 ± 4.6
	500	4.9	−2.2	6.6	4.7	98.6 ± 3.2	95.6 ± 2.5
	2000	5.1	4.5	3.2	8.3	92.8 ± 2.2	98.1 ± 3.2
	10	6.9	2.1	3.6	4.6	94.2 ± 2.5	99.6 ± 4.0
milnacipran	20	2.6	−4.3	4.0	−6.1	97.5 ± 4.7	92.4 ± 1.7
	500	4.1	−2.5	2.6	2.5	93.9 ± 3.0	94.5 ± 3.1
	2000	3.3	2.4	1.5	−3.0	94.5 ± 4.1	96.7 ± 5.1
	10	4.3	4.1	2.8	2.7	95.6 ± 3.5	107.9 ± 5.3
amitriptyline	20	2.6	3.3	7.9	6.3	101.4 ± 3.2	109.7 ± 4.9
	500	3.8	3.7	3.2	−5.3	95.7 ± 2.7	96.9 ± 4.5
	2000	3.0	−2.6	6.5	4.1	94.3 ± 4.8	98.7 ± 2.8
	10	5.4	3.4	2.7	3.4	97.7 ± 4.1	97.0 ± 3.0
fluvoxamine	20	2.6	5.7	4.5	2.7	96.2 ± 3.6	94.4 ± 1.7
	500	6.3	−5.2	3.7	−5.1	91.9 ± 2.7	94.4 ± 1.3
	2000	3.1	−3.2	2.7	0.6	95.7 ± 4.7	98.5 ± 2.4
	10	3.0	−1.4	4.1	3.3	95.5 ± 2.9	96.9 ± 4.7
o-desmethylvenlafaxine	20	2.9	2.9	3.9	−2.0	99.8 ± 3.6	95.2 ± 3.6
	500	4.8	4.2	6.2	2.7	94.6 ± 3.0	97.2 ± 5.1
	2000	1.7	3.7	4.4	−4.2	97.3 ± 2.6	95.2 ± 3.2
	10	5.6	−0.7	2.9	4.5	93.0 ± 4.0	93.8 ± 3.5
desmethylclomipramine	20	4.8	5.3	9.5	−6.9	95.4 ± 3.7	97.5 ± 4.9
	500	3.3	−2.7	5.2	2.1	102.5 ± 5.6	91.9 ± 2.5
	2000	2.8	3.1	7.4	−3.5	100.0 ± 4.2	94.2 ± 1.2
	20	3.6	2.6	2.6	10.9	96.5 ± 2.0	97.3 ± 1.3
venlafaxine	40	3.9	6.4	8.2	−2.6	98.2 ± 2.8	98.1 ± 3.1
	1000	2.8	3.0	3.6	5.3	97.8 ± 3.8	92.5 ± 4.8
	4000	4.6	5.5	4.5	1.7	94.3 ± 1.6	99.3 ± 4.2
	20	5.8	−3.2	5.3	−3.2	96.0 ± 3.4	97.5 ± 2.7
clomipramine	40	6.8	−4.9	4.2	−6.3	99.5 ± 1.7	100.5 ± 5.0
	1000	3.6	8.9	6.0	2.9	103.8 ± 3.5	98.2 ± 3.5
	4000	4.2	11.6	4.7	2.3	95.3 ± 4.4	97.4 ± 4.8
sertraline-d3	100	------	------	------	------	93.6 ± 3.9	96.8 ± 4.7
mirtazapine-d3	100	------	------	------	------	97.3 ± 3.5	98.2 ± 2.7
vortioxetine-d8	100	------	------	------	------	96.4 ± 3.6	98.7 ± 4.5
agomelatine-d6	100	------	------	------	------	98.2 ± 2.8	96.7 ± 3.0
bupropion-d9	100	------	------	------	------	103.5 ± 4.6	99.0 ± 2.5
mianserin-d3	100	------	------	------	------	93.7 ± 3.0	98.2 ± 3.7
citalopram-d6	100	------	------	------	------	99.5 ± 5.1	94.0 ± 1.6
paroxetine-d4	100	------	------	------	------	100.6 ± 2.2	97.5 ± 2.5
duloxetine-d7	100	------	------	------	------	96.7 ± 1.7	104.2 ± 6.8
fluoxetine-d5	200	------	------	------	------	98.2 ± 3.5	101.8 ± 0.9
norfluoxetine-d5	200	------	------	------	------	95.3 ± 2.8	99.4 ± 5
fluvoxamine-d3	200	------	------	------	------	99.1 ± 4.4	96.0 ± 4.2
clomipramine-d3	200	------	------	------	------	94.8 ± 4.8	97.7 ± 2.1
desmethylclomipramine-d3	200	------	------	------	------	93.9 ± 2.6	95.3 ± 3.9
desmethyldoxepin-d3	200	------	------	------	------	98.5 ± 3.7	98.8 ± 4.7
venlafaxine-d6	200	------	------	------	------	96.0 ± 4.5	98.4 ± 3.6
nortriptyline-d4	200	------	------	------	------	97.4 ± 3.0	102.6 ± 4.5
amitriptyline-d6	200	------	------	------	------	98.3 ± 3.8	97.2 ± 5.3
doxepine-d3	200	------	------	------	------	99.2 ± 2.6	95.8 ± 1.6
milnacipran-d5	200	------	------	------	------	96.2 ± 4.1	97.3 ± 2.8
o-desmethylvenlafaxine-d3	200	------	------	------	------	94.0 ± 2.8	104.6 ± 4.7
hydroxybupropione-d6	400	------	------	------	------	99.3 ± 5.9	94.2 ± 3.5
trazodone-d4	400	------	------	------	------	98.5 ± 5.5	100.2 ± 4.0

#### 3.2.4 Extraction recovery and matrix effect

In this study, although 23 antidepressants and active metabolites were simultaneously tested, the recovery and matrix effect of all analytes met the corresponding standards. The specific results are shown in Table 3. In terms of the recovery, it can be seen that the percentage recoveries of analytes and ISs ranged from 91.7% to 106.5% and 93.6%–103.5%, respectively. Meanwhile, we found that the recovery of all analytes and ISs was relatively stable, which also indicated that the extraction method we have chosen was suitable. As for the matrix effect, it was a key factor determining whether the established HPLC–MS/MS method can be applied in practice. Here, the percentage matrix effect of analytes and ISs ranged from 91.2% to 109.7% and 94.2%–104.6%, respectively. Overall, the matrix effect generated by endogenous substances has minimal impact on the accurate quantification of the analytes.

#### 3.2.5 Stability

Table 4 summarizes the stability data of the analytes in human serum at the QC level after storage. It was found that 23 antidepressants, active metabolites and ISs were stable in human serum under four different storage environments. The passing of stability experiment verification indicates that testing all analytes in real working scenarios will not affect the accuracy of the measurement results.

**TABLE 4:** Stability of analytes under various storage conditions (RE %, n = 4).

Drug	QC concentration	Room temperature	−70°C for 30 days	Freeze-thaw cycles	Autosampler stability	Dilution integrity	
						10-fold	50-fold
sertraline	8	3.4	2.9	3.9	1.9	------	------
	200	−4.1	−2.1	3.1	3.5	------	------
	800	5.2	6.2	−3.3	3.5	−3.5	2.8
mirtazapine	8	3.9	3.4	4.9	−4.6	------	------
	200	6.7	3.5	−1.8	3.7	------	------
	800	−2.8	1.9	4.2	2.8	3.2	−0.8
	8	3.0	4.1	−3.6	−0.9	------	------
vortioxetine	200	1.7	3.8	−3.1	3.2	------	------
	800	6.3	−2.9	−2.7	2.6	1.6	2.3
	8	4.2	3.3	3.5	−5.4	------	------
agomelatine	200	−7.9	−1.5	3.7	3.7	------	------
	800	4.6	2.6	3.8	−4.4	2.7	4.5
	8	3.7	2.3	3.9	2.5	------	------
bupropion	200	−6.4	3.4	4.6	6.7	------	------
	800	5.2	−3.8	−2.5	7.1	−3.4	1.0
	8	1.4	−2.5	6.3	−4.2	------	------
mianserin	200	3.3	2.8	−4.6	1.4	------	------
	800	−6.4	−3.6	−1.9	−2.9	2.8	2.6
	8	−1.9	4.1	4.8	5.2	------	------
escitalopram	200	−2.3	−3.8	−2.7	3.7	------	------
	800	−5.8	2.7	−4.5	3.1	3.3	−1.7
	8	2.9	1.3	4.9	−2.6	------	------
paroxetine	200	1.6	−1.9	−5.3	4.6	------	------
	800	1.9	2.4	−2.1	−2.8	4.5	5.2
	8	−2.2	3.4	−3.6	2.1	------	------
duloxetine	200	−3.6	3.0	3.3	0.7	------	------
	800	2.7	2.1	3.9	1.6	2.5	3.4
	20	2.5	−1.4	4.0	3.5	------	------
fluoxetine	500	−3.3	2.9	6.2	−4.2	------	------
	2000	4.9	−2.6	−2.8	1.8	−1.0	2.3
	20	2.4	2.8	−4.0	2.3	------	------
norfluoxetine	500	−4.3	−1.0	3.7	−3.5	------	------
	2000	2.9	4.3	4.2	−4.2	3.9	4.1
	20	4.7	2.6	5.2	1.6	------	------
hydrochloride	500	−3.5	2.3	−3.0	−1.9	------	------
	2000	−3.1	−4.8	33	2.0	2.2	1.3
	20	4.1	2.9	6.4	3.8	------	------
fluvoxamine	500	2.2	−1.7	−1.7	5.0	------	------
	2000	2.4	3.1	−2.7	−2.7	−3.5	1.8
	20	−2.7	−2.0	5.5	2.5	------	------
clomipramine	500	3.0	2.7	3.5	1.5	------	------
	2000	1.8	−2.9	3.9	3.0	4.9	5.9
	20	3.8	3.3	−2.2	2.6	------	------
desmethylclomipramine	500	2.5	−2.6	−3.7	3.7	------	------
	2000	3.5	−4.6	4.1	3.1	2.4	2.5
	20	−2.6	2.9	−3.42	−1.9	------	------
desmethyldoxepin	500	5.7	−1.5	3.6	1.1	------	------
	2000	4.9	4.6	3.2	3.5	−1.9	4.0
	20	−3.8	5.7	−4.8	−2.1	------	------
venlafaxine	500	4.4	3.8	−2.2	1.0	------	------
	2000	2.0	3.1	−2.1	0.5	3.1	2.5
	20	4.3	−2.8	0.8	−2.8	------	------
o-desmethylvenlafaxine	500	2.6	4.2	−3.9	3.6	------	------
	2000	−3.0	4.7	2.7	2.1	1.6	2.3
	20	−5.1	3.4	3.7	−4.6	------	------
doxepine	500	4.2	2.8	−4.6	4.1	------	------
	2000	3.6	−5.5	2.0	2.3	3.1	−0.8
	20	4.7	1.0	1.8	−1.3	------	------
milnacipran	500	1.9	3.5	−2.7	2.8	------	------
	2000	3.6	2.5	2.3	2.6	4.2	−2.7
	20	4.4	5.6	−5.8	4.5	------	------
amitriptyline	500	−5.2	6.2	2.4	3.4	------	------
	2000	4.8	−2.7	4.3	−3.0	4.3	1.6
	20	3.1	3.4	−1.7	2.6	------	------
nortriptyline hydrochloride	500	2.4	3.6	2.5	4.2	------	------
	2000	1.5	−2.9	3.2	−1.7	3.5	−1.6
	40	−5.7	1.6	1.0	3.8	------	------
hydroxybupropione	1000	3.9	2.5	−2.6	−1.8	------	------
	4000	4.6	4.0	3.8	2.0	2.7	2.1
	40	−3.4	−3.6	2.9	−0.9	------	------
trazodone	1000	2.0	2.4	4.0	0.7	------	------
	4000	1.9	1.7	3.8	1.8	1.0	1.6

#### 3.2.6 Dilution integrity

The TDM indicators for antidepressants recommend steady-state trough concentrations, but some patients may have serum drug concentrations higher than the upper limit of the standard curve, including patients taking multiple drugs simultaneously, patients giving blood samples after medication, patients increasing the dosage without authorization and patients in special populations. Therefore, it is necessary to dilute the serum of these patients to achieve accurate measurement. In this paper, dilution integrity was evaluated by diluting serum samples higher than ULOQ with blank serum to HQC levels, and the results are exhibited in Table 4. We found that the accuracy and the precision of dilution integrity were better than ±15%. Therefore, serum samples of antidepressants in the clinic with a concentration higher than ULOQ can be diluted with blank human serum before processing.

### 3.3 Application

The TDM index for antidepressants is steady-state trough concentration, and a reasonable blood collection plan can ensure that TDM results are used to guide the adjustment of medication regimens for patients with depression. According to relevant guidelines and expert consensus at home and abroad, we have summarized the recommended concentration range and blood collection plan for antidepressants, as shown in Table 5; (Hiemke et al., 2017).

**TABLE 5:** Recommended concentration range and blood collection time for antidepressants and active metabolites.

Drug	Recommended range	Warning value	Blood collection time
sertraline	10–150 ng/mL	300 ng/mL	take medication at least 5 times and collect blood before the next medication
fluoxetine + norfluoxetine	120–500 ng/mL	1,000 ng/mL	half life of 4–6 days, reaching steady state after 20 days of medication, blood collection before medication the next morning
escitalopram	15–80 ng/mL	160 ng/mL	take medication at least 5 times and collect blood before the next medication
fluvoxamine	60–230 ng/mL	500 ng/mL	take medication at least 5 times and collect blood before the next medication
paroxetine	20–65 ng/mL	120 ng/mL	take medication at least 5 times and collect blood before the next medication
Venlafaxine + o-desmethylvenlafaxine	10–400 ng/mL	800 ng/mL	take medication at least 5 times and collect blood before the next medication
duloxetine	30–120 ng/mL	240 ng/mL	take medication at least 5 times and collect blood before the next medication
mirtazapine	30–80 ng/mL	160 ng/mL	take medication at least 5 times and collect blood before the next medication
amitriptyline + nortriptyline	80–200 ng/mL	300 ng/mL	take medication at least 5 times and collect blood before the next medication
doxepine+desmethyldoxepin	50–150 ng/mL	300 ng/mL	take medication at least 5 times and collect blood before the next medication
bupropion + hydroxybupropione	850–1,500 ng/mL	2000 ng/mL	take medication at least 5 times and collect blood before the next medication
trazodone	700–1,000 ng/mL	1,200 ng/mL	take medication at least 5 times and collect blood before the next medication
milnacipran	10–40 ng/mL	80 ng/mL	take medication at least 5 times and collect blood before the next medication
mianserin	15–70 ng/mL	140 ng/mL	take medication at least 5 times and collect blood before the next medication
clomipramine + desmethylclomipramine	230–450 ng/mL	450 ng/mL	half life of 1–3 days, reaching steady state after 10 days of medication, blood collection before medication the next morning
vortioxetine	10–40 ng/mL	80 ng/mL	half life of 57–66 h, reaching steady state after 13 days of medication, blood collection before medication the next morning
agomelatine	7–300 ng/mL (1–2 h after 50 mg)	600 ng/mL	After taking the medication at least 5 times, blood samples should be collected between 1 and 2 h after taking the medication to measure C_max_

First, we found that the HPLC–MS/MS method can quickly and accurately detect the serum drug concentration of antidepressants and active metabolites. The calibration curve and QC sample of each antidepressant were quantified. Therefore, the established HPLC–MS/MS method can be used for pharmacokinetic studies and TDM of antidepressant drugs. Second, the proportion of steady-state trough concentrations of antidepressants that are not within the recommended treatment range can reach 37%–62% according to the TDM results of this study. The dose-related concentration reference range is a landmark reference range for identifying patients with abnormal serum drug concentrations. When conducting TDM work, the measured serum drug concentration reported by the TDM laboratory should be compared with the theoretical values recommended in the guidelines. When the patient’s serum drug concentration falls within the expected dose-related reference concentration range, it can be considered “normal,” which means the concentration matches the prescribed dose. Concentrations above or below the expected range indicate potential abnormalities, such as partial noncompliance, drug–drug interactions, genetic polymorphisms of drug-metabolizing enzymes or diseases of organs associated with drug elimination. In summary, once abnormal conditions are observed, TDM clinical pharmacology opinions should analyze possible influencing factors and clarify their causes. Third, some antidepressants, such as venlafaxine, bupropion, fluoxetine, amitriptyline, doxepine and clomipramine, can produce metabolites with similar or different pharmacokinetic characteristics from the parent drug through biotransformation by phase I metabolic enzymes. In this case, the sum of the concentrations of the parent drug and active metabolites may be more meaningful for guiding patient dosage adjustments. Compared with immunoassay and HPLC, the HPLC–MS/MS method established in this article can simultaneously determine the concentrations of 23 commonly used clinical antidepressants and their active metabolites, which is crucial for precise medication in patients. This can also explain the situation where some patients have low concentrations of the parent drug but still have good therapeutic effects, which may be related to the concentration of active metabolites, such as venlafaxine and bupropion. Finally, we followed up with 100 patients through communication with doctors and found that adjusting the medication regimen based on TDM could significantly improve the efficacy or reduce adverse reactions in patients. We found that 89% of patients had serum drug concentrations within the recommended treatment range after TDM intervention. In summary, it is feasible to use the HPLC–MS/MS method to determine serum drug concentrations and guide dosage adjustment in patients with depression.

## 4 Conclusion

Through TDM, we found that: 1) conducting TDM targeting antidepressant drugs had clear indications and significant importance; 2) the steady-state trough concentration of most depressed patients was not within the recommended treatment range (Eichentopf et al., 2022; Florio et al., 2017); 3) for antidepressants with active metabolites, it was more meaningful to evaluate the relationship between dosage and efficacy using the total concentration of the parent drug and metabolites (Funk et al., 2022). Therefore, this method is suitable for TDM of 23 antidepressants and active metabolites and potential pharmacokinetics study.

In this study, we successfully established an HPLC–MS/MS method capable of simultaneously detecting the serum drug concentrations of commonly used antidepressants and their active metabolites in clinical practice, followed by a systematic validation process. This HPLC–MS/MS method demonstrated remarkable characteristics, including strong specificity, good stability, high sensitivity, and an appropriate retention time. Ultimately, this method was effectively applied to the TDM of antidepressant drugs, effectively overcoming the limitations of immunological and HPLC methods.

Through TDM, several key findings emerged: 1) Conducting TDM for antidepressant drugs has clear indications and is of significant importance. 2) The steady - state trough concentration of most depressed patients did not fall within the recommended treatment range (Eichentopf et al., 2022; Florio et al., 2017). 3) For antidepressants with active metabolites, evaluating the relationship between dosage and efficacy using the total concentration of the parent drug and metabolites is more meaningful (Funk et al., 2022).

Notably, while the HPLC-MS/MS method we developed offers high-quality detection capabilities, it should be acknowledged that, due to the use of MS/MS detection, it is not a cost-effective approach. However, its ability to accurately detect 23 antidepressants and their active metabolites, along with its potential for pharmacokinetics studies, justifies its application. This method is well-suited for TDM of these substances, and despite the cost factor, it provides valuable insights into the use of antidepressant medications. Future research could focus on exploring ways to optimize the cost-effectiveness of this method without sacrificing its high-performance capabilities.

In the introduction and throughout the discussion, we have emphasized the significance of this method in addressing the limitations of existing techniques. The method’s potential for contributing to more precise TDM and understanding of antidepressant pharmacology underscores its importance in the field, despite the associated cost implications.

## Data Availability

The original contributions presented in the study are included in the article/Supplementary Material, further inquiries can be directed to the corresponding author.
