# Supplementary material for: High-performance liquid chromatography–tandem mass spectrometry for simultaneous determination of 23 antidepressants and active metabolites in human serum and its application in therapeutic drug monitoring
Source: Front Pharmacol. 2025 Mar 27;16:1531496. doi: 10.3389/fphar.2025.1531496 (PMC11983492; doi:10.3389/fphar.2025.1531496)
Supplement: Supplementary file 1 [file Image1.pdf]

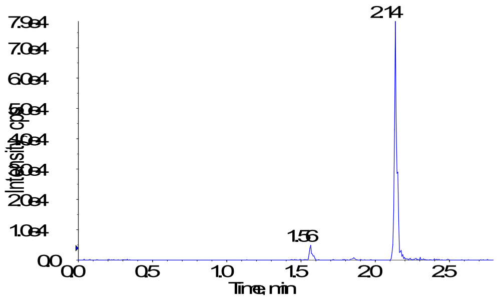

Sertraline-LLOQ

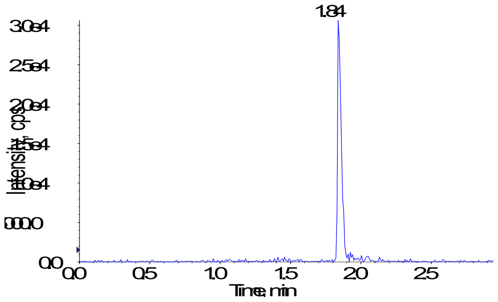

Escitralopram-LLOQ

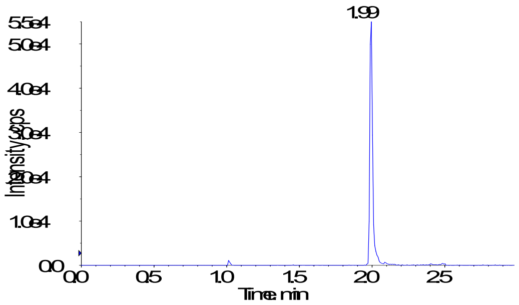

Fluvoxamine-LLOQ

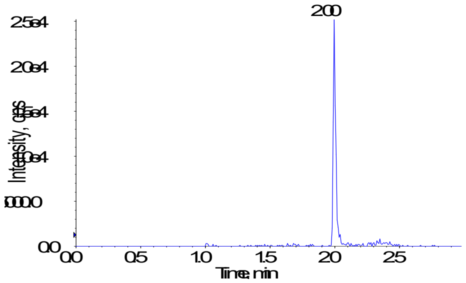

Paroxetine-LLOQ

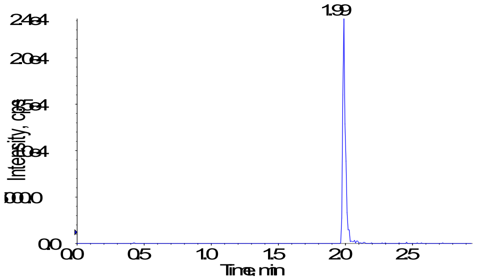

Duloxetine-LLOQ

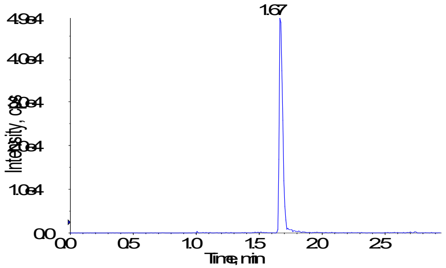

Milnacipran-LLOQ

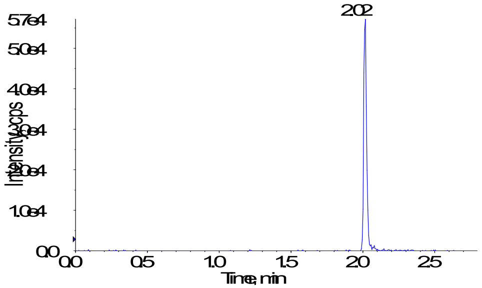

Fuoxetine-LLOQ

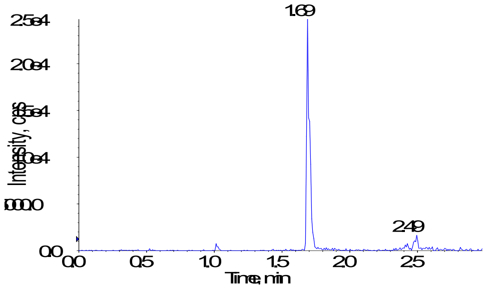

Venlafaxine-LLOQ

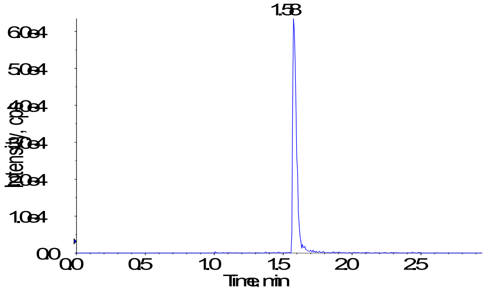

O-desmethylvenlafaxine-LLOQ

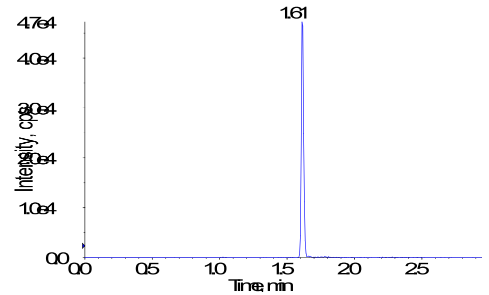

Mirtazapine-LLOQ

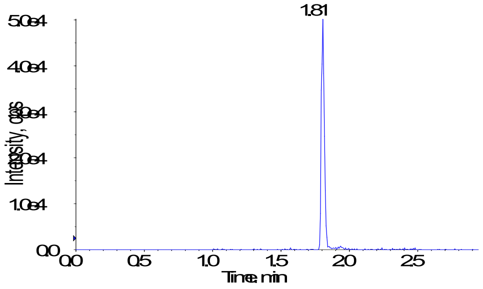

Trazodone-LLOQ

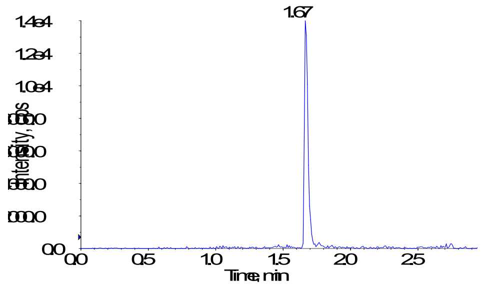

Bupropion-LLOQ

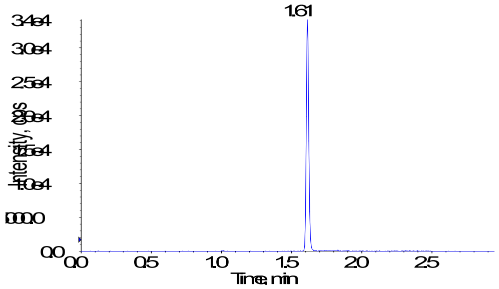

Hydroxybupropion-LLOQ

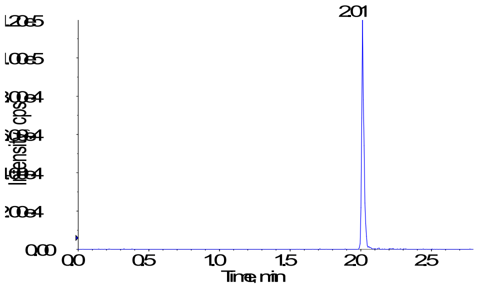

Norfluoxetine-LLOQ

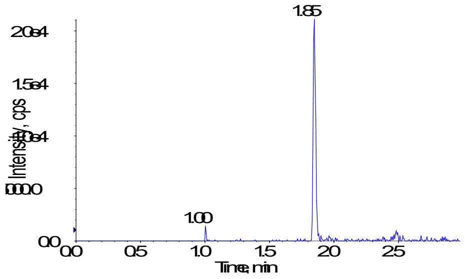

Mianserin-LLOQ

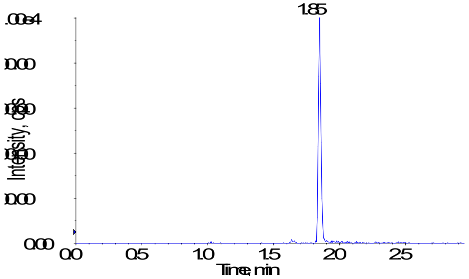

Nordoxepin-LLOQ

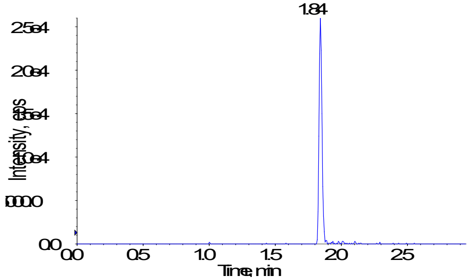

Doxepin-LLOQ

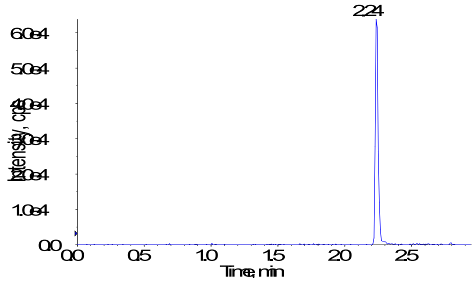

Agomelatine-LLOQ

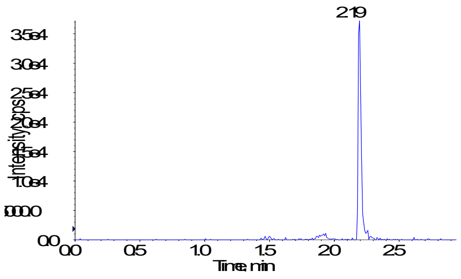

Vortioxetine-LLOQ

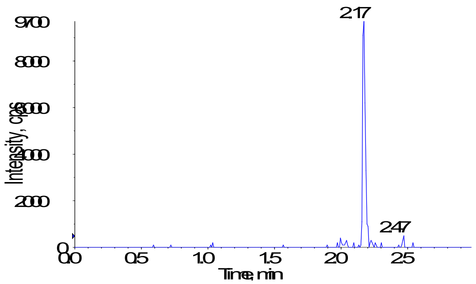

Clomipramine -LLOQ

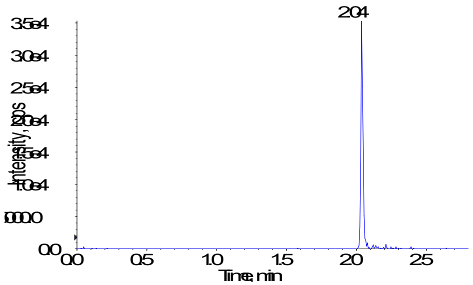

Amitriptyline-LLOQ

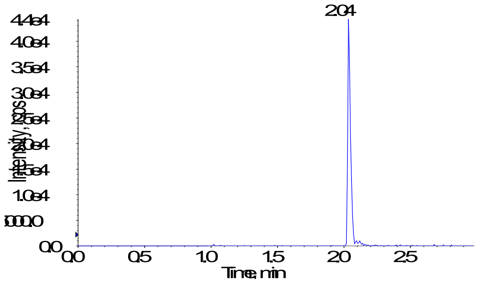

Nortriptyline-LLOQ

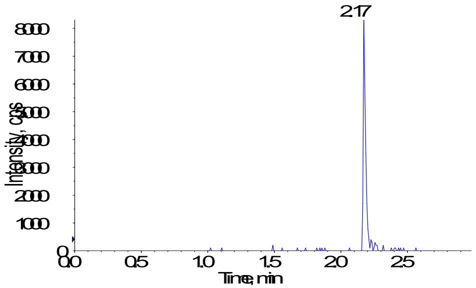

Norclomipramine-LLOQ

Supplementary figure1 Chromatograms of each analyte at the LLOQ level
